# 7-Diethyl­amino-2-oxo-2*H*-chromene-3-carbaldehyde

**DOI:** 10.1107/S160053681102294X

**Published:** 2011-06-18

**Authors:** Hong-Da Li, Bing-Zhu Yin

**Affiliations:** a Key Laboratory of Natural Resources of Changbai Mountain & Functional Molecules (Yanbian University), Ministry of Education, Yanji 133002, People’s Republic of China

## Abstract

In the title compound, C_14_H_15_NO_3_, all non-H atoms except for those of the methyl and the disordered ethyl groupare approximately co-planar, the largest deviation from the mean plane being 0.0223 (13) Å at the N atom. In the crystal, the packing of mol­ecules through weak inter­molecular C—H⋯O hydrogen-bonding inter­actions leads to the formation of layers parallel to *bc* plane. Within these layers, there exist slipped π–π stacking inter­actions between symmetry-related fused rings [centroid–centroid distances = 3.527 (3) and 3.554 (3), slippage = 0.988 and 1.011 Å, respectively]. One ethyl group is disordered over two sets of sites with site-occupation factors of 0.54 and 0.46.

## Related literature

For background to the title compound, an organic inter­mediate and a fluorescent probe for cyanide and amino acids, see: Kim *et al.* (2010 ▶). For electronic and photonic applications of coumarins, see: Murray *et al.* (1982 ▶). For the synthesis, see: Wu *et al.* (2007 ▶).
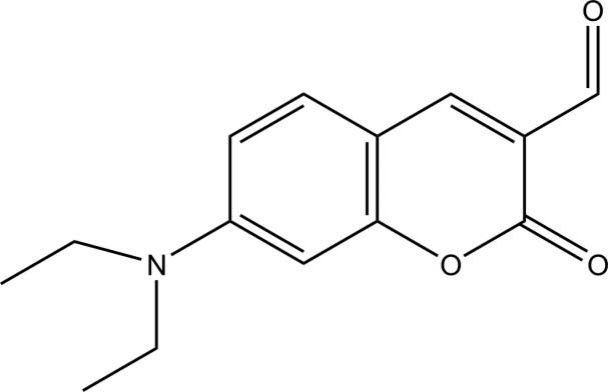


## Experimental

### 

#### Crystal data

C_14_H_15_NO_3_
*M* *_r_* = 245.27
Monoclinic, 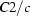

*a* = 25.488 (17) Å
*b* = 7.844 (6) Å
*c* = 12.599 (12) Å
β = 92.39 (3)°
*V* = 2517 (3) Å^3^
*Z* = 8
Mo *K*α radiation
μ = 0.09 mm^−1^
*T* = 295 K
0.41 × 0.39 × 0.21 mm

#### Data collection

Rigaku R-AXIS RAPID diffractometer
Absorption correction: multi-scan (*ABSCOR*; Higashi, 1995 ▶) *T*_min_ = 0.963, *T*_max_ = 0.981
11514 measured reflections
2850 independent reflections
1724 reflections with *I* > 2σ(*I*)
*R*_int_ = 0.033

#### Refinement

*R*[*F* ^2^ > 2σ(*F* ^2^)] = 0.048
*wR*(*F* ^2^) = 0.144
*S* = 1.06
2850 reflections
184 parameters
1 restraint
H-atom parameters constrained
Δρ_max_ = 0.14 e Å^−3^
Δρ_min_ = −0.15 e Å^−3^

### 

Data collection: *RAPID-AUTO* (Rigaku, 1998 ▶); cell refinement: *RAPID-AUTO*; data reduction: *CrystalStructure* (Rigaku, 2002 ▶); program(s) used to solve structure: *SHELXS97* (Sheldrick, 2008 ▶); program(s) used to refine structure: *SHELXL97* (Sheldrick, 2008 ▶); molecular graphics: *ORTEPIII* (Burnett & Johnson, 1996 ▶) and *ORTEP-3 for Windows* (Farrugia, 1997 ▶); software used to prepare material for publication: *SHELXL97*.

## Supplementary Material

Crystal structure: contains datablock(s) global, I. DOI: 10.1107/S160053681102294X/dn2698sup1.cif

Structure factors: contains datablock(s) I. DOI: 10.1107/S160053681102294X/dn2698Isup2.hkl

Supplementary material file. DOI: 10.1107/S160053681102294X/dn2698Isup3.cml

Additional supplementary materials:  crystallographic information; 3D view; checkCIF report

## Figures and Tables

**Table 1:** Hydrogen-bond geometry (Å, °)

*D*—H⋯*A*	*D*—H	H⋯*A*	*D*⋯*A*	*D*—H⋯*A*
C8—H8⋯O3^i^	0.93	2.58	3.367 (4)	143
C9—H9⋯O1^ii^	0.93	2.55	3.432 (3)	158
C13—H13*B*⋯O2^iii^	0.97	2.53	3.388 (3)	147

Symmetry codes: (i) 
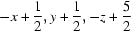
; (ii) 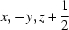; (iii) 
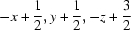
.

**Fig. 1.**
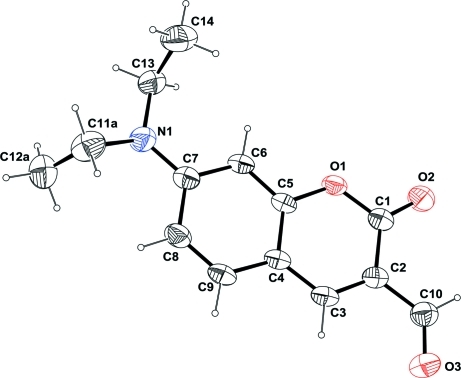
The crystal structure of the title compound, with the atom numbering scheme. Displacement ellipsoids of non-H atoms are drawn at the 30% probalility level. H atoms are shown as small spheres of arbitrary radii. Only the major component of the disordered ethyl is represented for clarity.

## supplementary crystallographic information

### Comment

Coumarins are an important class of naturally occurring and synthetic compounds, which have been extentively investigated for electronic and photonic applications (Murray *et al.*, 1982). Herein, we reported the crystal structure of the title compound, an important organic intermediate and a fluorescent probe for cyanide and amino acids (Kim *et al.*, 2010).

The molecule of title compound formed by two fused rings is mainly planar with the exception of the methyl and disordered ethyl group (Fig. 1). The weak intermolcular C—H···O hydrogen bonds (Table 1) link the molecules into layers parallel to the (100) plane. Futhermore, slipped π-π stacking occurs between symetry related fused rings within the layers (Table 2)

### Experimental

The title compound was prepared according to the literature (Wu *et al.*, 2007). Single crystals suitable for X-ray diffraction were prepared by recrystallization from mixture of dichloromethane and petroleum (60–90 °C).

### Refinement

Carbon-bound H-atoms were placed in calculated positions (C—H 0.93 to 0.97 Å) and were included in the refinement in the riding model with *U*_iso_(H) = 1.2 or 1.5 *U*_eq_(C_methyl_).

One of the ethyl group is disordered over two positions with a site occupancy in the ratio 0.54/0.46. The refinement of the disordered moieties was carried out using the PART instruction and restraining them to have identical geometry with the SAME instruction available in SHELXL-97 (Sheldrick, 2008)

### Crystal data

**Table:**

C_14_H_15_NO_3_	*F*(000) = 1040
*M**_r_* = 245.27	*D*_x_ = 1.295 Mg m^−^^3^
Monoclinic, *C*2/*c*	Mo *K*α radiation, λ = 0.71073 Å
Hall symbol: -C 2yc	Cell parameters from 6542 reflections
*a* = 25.488 (17) Å	θ = 3.2–27.5°
*b* = 7.844 (6) Å	µ = 0.09 mm^−^^1^
*c* = 12.599 (12) Å	*T* = 295 K
β = 92.39 (3)°	Block, brown
*V* = 2517 (3) Å^3^	0.41 × 0.39 × 0.21 mm
*Z* = 8	

### Data collection

**Table:**

Rigaku R-AXIS RAPID diffractometer	2850 independent reflections
Radiation source: fine-focus sealed tube	1724 reflections with *I* > 2σ(*I*)
graphite	*R*_int_ = 0.033
ω scans	θ_max_ = 27.5°, θ_min_ = 3.2°
Absorption correction: multi-scan (*ABSCOR*; Higashi, 1995)	*h* = −32→32
*T*_min_ = 0.963, *T*_max_ = 0.981	*k* = −9→10
11514 measured reflections	*l* = −16→16

### Refinement

**Table:**

Refinement on *F*^2^	Primary atom site location: structure-invariant direct methods
Least-squares matrix: full	Secondary atom site location: difference Fourier map
*R*[*F*^2^ > 2σ(*F*^2^)] = 0.048	Hydrogen site location: inferred from neighbouring sites
*wR*(*F*^2^) = 0.144	H-atom parameters constrained
*S* = 1.06	*w* = 1/[σ^2^(*F*_o_^2^) + (0.0674*P*)^2^ + 0.3544*P*] where *P* = (*F*_o_^2^ + 2*F*_c_^2^)/3
2850 reflections	(Δ/σ)_max_ < 0.001
184 parameters	Δρ_max_ = 0.14 e Å^−^^3^
1 restraint	Δρ_min_ = −0.15 e Å^−^^3^

### Special details

**Table:**

Experimental. (See detailed section in the paper)
Geometry. All esds (except the esd in the dihedral angle between two l.s. planes) are estimated using the full covariance matrix. The cell esds are taken into account individually in the estimation of esds in distances, angles and torsion angles; correlations between esds in cell parameters are only used when they are defined by crystal symmetry. An approximate (isotropic) treatment of cell esds is used for estimating esds involving l.s. planes.
Refinement. Refinement of *F*^2^ against ALL reflections. The weighted *R*-factor *wR* and goodness of fit *S* are based on *F*^2^, conventional *R*-factors *R* are based on *F*, with *F* set to zero for negative *F*^2^. The threshold expression of *F*^2^ > σ(*F*^2^) is used only for calculating *R*-factors(gt) *etc*. and is not relevant to the choice of reflections for refinement. *R*-factors based on *F*^2^ are statistically about twice as large as those based on *F*, and *R*- factors based on ALL data will be even larger.

### Fractional atomic coordinates and isotropic or equivalent isotropic displacement parameters (Å^2^)

**Table:**

	*x*	*y*	*z*	*U*_iso_*/*U*_eq_	Occ. (<1)
O1	0.24299 (4)	0.00447 (14)	0.88750 (8)	0.0556 (3)	
O2	0.17217 (5)	−0.12688 (18)	0.82505 (10)	0.0787 (4)	
O3	0.12022 (6)	−0.24463 (18)	1.11710 (12)	0.0874 (5)	
N1	0.40226 (6)	0.2780 (2)	0.99586 (12)	0.0747 (5)	
C1	0.19772 (6)	−0.0872 (2)	0.90386 (13)	0.0562 (4)	
C2	0.18575 (6)	−0.12332 (19)	1.01323 (12)	0.0533 (4)	
C3	0.21890 (6)	−0.0707 (2)	1.09396 (12)	0.0546 (4)	
H3	0.2105	−0.0940	1.1636	0.066*	
C4	0.26540 (6)	0.01794 (19)	1.07523 (11)	0.0503 (4)	
C5	0.27674 (6)	0.05510 (19)	0.96975 (11)	0.0478 (4)	
C6	0.32073 (6)	0.1413 (2)	0.94200 (12)	0.0533 (4)	
H6	0.3261	0.1652	0.8710	0.064*	
C7	0.35790 (6)	0.1938 (2)	1.02108 (13)	0.0578 (4)	
C8	0.34695 (7)	0.1572 (2)	1.12853 (13)	0.0636 (5)	
H8	0.3707	0.1906	1.1826	0.076*	
C9	0.30257 (7)	0.0747 (2)	1.15318 (12)	0.0613 (4)	
H9	0.2963	0.0546	1.2243	0.074*	
C10	0.13764 (7)	−0.2172 (2)	1.03121 (16)	0.0679 (5)	
H10	0.1189	−0.2592	0.9719	0.081*	
C11A	0.4515 (3)	0.2676 (10)	1.0761 (7)	0.086 (2)	0.46
H11A	0.4478	0.1756	1.1267	0.103*	0.46
H11B	0.4836	0.2509	1.0387	0.103*	0.46
C12A	0.4513 (2)	0.4378 (8)	1.1303 (5)	0.1030 (17)	0.46
H12A	0.4539	0.5266	1.0784	0.154*	0.46
H12B	0.4807	0.4448	1.1805	0.154*	0.46
H12C	0.4193	0.4510	1.1669	0.154*	0.46
C11B	0.4390 (2)	0.3468 (8)	1.0753 (5)	0.0750 (15)	0.54
H11C	0.4204	0.3835	1.1370	0.090*	0.54
H11D	0.4570	0.4445	1.0469	0.090*	0.54
C12B	0.4784 (2)	0.2088 (8)	1.1068 (5)	0.125 (2)	0.54
H12D	0.4602	0.1130	1.1354	0.188*	0.54
H12E	0.5031	0.2527	1.1595	0.188*	0.54
H12F	0.4966	0.1734	1.0455	0.188*	0.54
C13	0.41299 (8)	0.3169 (3)	0.88577 (15)	0.0742 (5)	
H13A	0.4374	0.4116	0.8847	0.089*	
H13B	0.3806	0.3533	0.8495	0.089*	
C14	0.43552 (9)	0.1696 (3)	0.8251 (2)	0.1025 (8)	
H14A	0.4692	0.1394	0.8563	0.154*	
H14B	0.4393	0.2024	0.7524	0.154*	
H14C	0.4123	0.0735	0.8280	0.154*	

### Atomic displacement parameters (Å^2^)

**Table:**

	*U*^11^	*U*^22^	*U*^33^	*U*^12^	*U*^13^	*U*^23^
O1	0.0602 (6)	0.0714 (7)	0.0356 (6)	−0.0004 (6)	0.0062 (4)	0.0006 (5)
O2	0.0830 (8)	0.1019 (10)	0.0503 (7)	−0.0158 (7)	−0.0056 (6)	0.0026 (7)
O3	0.0943 (10)	0.0941 (10)	0.0756 (9)	−0.0150 (8)	0.0259 (8)	0.0101 (7)
N1	0.0630 (8)	0.1053 (12)	0.0559 (9)	−0.0141 (8)	0.0021 (7)	0.0042 (8)
C1	0.0627 (9)	0.0599 (10)	0.0459 (9)	0.0078 (8)	0.0030 (7)	0.0007 (7)
C2	0.0613 (9)	0.0508 (9)	0.0486 (9)	0.0078 (8)	0.0114 (7)	0.0042 (7)
C3	0.0697 (10)	0.0563 (9)	0.0387 (8)	0.0110 (8)	0.0125 (7)	0.0036 (7)
C4	0.0612 (9)	0.0544 (9)	0.0357 (8)	0.0106 (7)	0.0091 (6)	0.0020 (6)
C5	0.0565 (8)	0.0530 (9)	0.0342 (7)	0.0129 (7)	0.0044 (6)	−0.0019 (6)
C6	0.0602 (9)	0.0646 (10)	0.0358 (8)	0.0082 (8)	0.0089 (6)	0.0018 (7)
C7	0.0604 (9)	0.0651 (10)	0.0479 (9)	0.0078 (8)	0.0044 (7)	0.0016 (7)
C8	0.0707 (11)	0.0787 (12)	0.0408 (9)	0.0001 (9)	−0.0031 (7)	0.0005 (8)
C9	0.0784 (11)	0.0727 (11)	0.0329 (8)	0.0061 (9)	0.0046 (7)	0.0034 (7)
C10	0.0754 (11)	0.0657 (11)	0.0631 (12)	0.0018 (9)	0.0103 (9)	0.0037 (9)
C11A	0.069 (4)	0.099 (6)	0.091 (4)	0.013 (4)	0.007 (3)	0.022 (4)
C12A	0.093 (4)	0.121 (5)	0.094 (4)	−0.022 (3)	−0.009 (3)	−0.008 (4)
C11B	0.065 (3)	0.086 (4)	0.073 (3)	−0.015 (3)	−0.013 (2)	0.005 (3)
C12B	0.083 (3)	0.152 (5)	0.137 (5)	0.036 (3)	−0.040 (3)	0.021 (4)
C13	0.0713 (11)	0.0878 (13)	0.0643 (11)	−0.0101 (10)	0.0123 (9)	0.0046 (10)
C14	0.0940 (15)	0.1172 (19)	0.0991 (18)	0.0080 (14)	0.0368 (13)	−0.0051 (15)

### Geometric parameters (Å, °)

**Table:**

O1—C5	1.3774 (19)	C9—H9	0.9300
O1—C1	1.382 (2)	C10—H10	0.9300
O2—C1	1.205 (2)	C11A—C12A	1.500 (8)
O3—C10	1.206 (2)	C11A—H11A	0.9700
N1—C7	1.358 (2)	C11A—H11B	0.9700
N1—C11B	1.446 (6)	C12A—H12A	0.9600
N1—C13	1.457 (3)	C12A—H12B	0.9600
N1—C11A	1.581 (8)	C12A—H12C	0.9600
C1—C2	1.452 (3)	C11B—C12B	1.517 (7)
C2—C3	1.359 (2)	C11B—H11C	0.9700
C2—C10	1.456 (3)	C11B—H11D	0.9700
C3—C4	1.402 (2)	C12B—H12D	0.9600
C3—H3	0.9300	C12B—H12E	0.9600
C4—C5	1.402 (2)	C12B—H12F	0.9600
C4—C9	1.408 (2)	C13—C14	1.512 (3)
C5—C6	1.367 (2)	C13—H13A	0.9700
C6—C7	1.408 (2)	C13—H13B	0.9700
C6—H6	0.9300	C14—H14A	0.9600
C7—C8	1.423 (2)	C14—H14B	0.9600
C8—C9	1.351 (2)	C14—H14C	0.9600
C8—H8	0.9300		
			
C5—O1—C1	122.47 (12)	O3—C10—C2	125.00 (19)
C7—N1—C11B	122.7 (3)	O3—C10—H10	117.5
C7—N1—C13	121.03 (15)	C2—C10—H10	117.5
C11B—N1—C13	116.1 (3)	C12A—C11A—N1	103.2 (5)
C7—N1—C11A	118.2 (3)	C12A—C11A—H11A	111.1
C13—N1—C11A	116.3 (3)	N1—C11A—H11A	111.1
O2—C1—O1	115.93 (15)	C12A—C11A—H11B	111.1
O2—C1—C2	127.14 (17)	N1—C11A—H11B	111.1
O1—C1—C2	116.93 (14)	H11A—C11A—H11B	109.1
C3—C2—C1	120.15 (16)	N1—C11B—C12B	108.5 (5)
C3—C2—C10	122.59 (16)	N1—C11B—H11C	110.0
C1—C2—C10	117.26 (16)	C12B—C11B—H11C	110.0
C2—C3—C4	121.88 (14)	N1—C11B—H11D	110.0
C2—C3—H3	119.1	C12B—C11B—H11D	110.0
C4—C3—H3	119.1	H11C—C11B—H11D	108.4
C3—C4—C5	118.13 (14)	C11B—C12B—H12D	109.5
C3—C4—C9	126.03 (14)	C11B—C12B—H12E	109.5
C5—C4—C9	115.84 (15)	H12D—C12B—H12E	109.5
C6—C5—O1	116.32 (13)	C11B—C12B—H12F	109.5
C6—C5—C4	123.28 (14)	H12D—C12B—H12F	109.5
O1—C5—C4	120.40 (15)	H12E—C12B—H12F	109.5
C5—C6—C7	119.92 (15)	N1—C13—C14	114.33 (19)
C5—C6—H6	120.0	N1—C13—H13A	108.7
C7—C6—H6	120.0	C14—C13—H13A	108.7
N1—C7—C6	121.26 (15)	N1—C13—H13B	108.7
N1—C7—C8	121.26 (16)	C14—C13—H13B	108.7
C6—C7—C8	117.48 (16)	H13A—C13—H13B	107.6
C9—C8—C7	120.99 (16)	C13—C14—H14A	109.5
C9—C8—H8	119.5	C13—C14—H14B	109.5
C7—C8—H8	119.5	H14A—C14—H14B	109.5
C8—C9—C4	122.47 (15)	C13—C14—H14C	109.5
C8—C9—H9	118.8	H14A—C14—H14C	109.5
C4—C9—H9	118.8	H14B—C14—H14C	109.5

### Hydrogen-bond geometry (Å, °)

**Table:**

*D*—H···*A*	*D*—H	H···*A*	*D*···*A*	*D*—H···*A*
C8—H8···O3^i^	0.93	2.58	3.367 (4)	143.
C9—H9···O1^ii^	0.93	2.55	3.432 (3)	158.
C13—H13B···O2^iii^	0.97	2.53	3.388 (3)	147.

Symmetry codes: (i) −*x*+1/2, *y*+1/2, −*z*+5/2; (ii) *x*, −*y*, *z*+1/2; (iii) −*x*+1/2, *y*+1/2, −*z*+3/2.

### Table 2 Table 2 π-π stacking interactions (Å,°)

Cg1 is the centroid of the O1—C5 ring.Cg2 is the centroid of the C4—C9 ring

**Table:**

CgI	CgJ	CgI···CgJ^a^	CgI···P(J)^b^	CgJ···P(I)^c^	Slippage
Cg1	Cg1^iv^	3.527 (3)	-3.3856 (6)	-3.3856 (6)	0.988
Cg1	Cg2^iv^	3.554 (3)	3.4110 (6)	3.4044 (6)	1.011

Symmetry codes: (iv)1/2-x,-1/2-y,2-z Notes:a : Distance between centroidsb : Perpendicular distance of CgI on ring plan Jc : Perpendicular distance of CgJ on ring plan ISlippage = vertical displacement between ring centroids.
